# The New York Institute of Stomatology

**Published:** 1896-04

**Authors:** 

**Affiliations:** The New York Institute of Stomatology


### THE NEW YORK INSTITUTE OF STOMATOLOGY.

A meeting of the Institute was held at the Polyclinic College and Hospital, 216 East Thirty-fourth Street, Tuesday evening, February 4, 1896, at eight o'clock.

The vice-president, Dr. C. D. Cook, presided, and introduced Dr. R. H. M. Dawbarn, professor of Surgery in the Polyclinic College and Hospital, who gave a clinical lecture and surgical demonstrations, as follows:

*Dr. Dawbarn.*—I notice that the cards of invitation announce a large number of demonstrations, and in order not to disappoint you I have brought some living subjects.

This young man, twenty-seven years of age, developed a sarcoma of the nasal pharynx. Its attachment was partly from the middle turbinated bone and partly from the pharynx at the base of the skull. It was so extremely vascular—bleeding freely on the slightest touch—that another surgeon who was asked to operate on it declined to do so because he thought the patient might die under the operation. Before he came to my hands Dr. Lederman had made thirteen injections of antitoxine, prepared from the streptococcus of erysipelas and the bacillus prodigiosus. By the time he reached the thirteenth injection he declined to go on, thinking there would be a funeral; and, indeed, they had wofully sapped his strength. There had been absolutely no benefit from this treatment; and there is hardly a doctor in New York now who has any confidence in injectional treatment save for the variety of sarcoma known as spindle-celled, and even that is *sub judice.* In the giant-cell and the round-cell variety antitoxine treatment proves absolutely useless, and it is a question whether in any other variety of sarcoma any better results are obtained by this method than from the injection of picric acid or of pyoktanin or methylene-blue. In this case the question arose whether I had a right to use the knife at all, because he bled so profusely. I started by tying both of the external carotids. You will notice that his neck shows a scar where I did that on each side. The operation was done last June, just before I went to Europe, and when I returned, in September, I found to my great delight that in the interval the tumor had shrunk to about a quarter of its former size from having almost its entire blood-supply cut off by ligation. If the circulation would only remain in that diminished condition we could expect a permanent shrinkage of the abnormal growth. The tumor remained small for two or three months further, and he said he felt better than he had for two years. Then it began to grow again from increased blood-supply, and it became a question of its removal. He consented to have that done as offering the only chance for his life. It was a desperate operation. I have never had quite as bloody a one, and that was a considerable surprise, because after having ligated the carotids we would not expect it. The hemorrhage was doubtless due to the fact that collateral circulation by anastomosis had been completely set up. The operation was done in this room three weeks ago. I will illuminate the mouth with this small electric light and show you the cavity. The incision runs from the prominence of the malar bone horizontally just below the orbit to the nose, thence along the naso-facial groove to the middle line, splitting the centre of the upper lip, and from this point along the roof of the mouth, at its centre, back to the soft palate, thence between the hard and soft palate on the affected side until the cheek is reached. The bone was partly sawn and partly chiselled along this whole length. The lower plate of the orbit was carefully preserved to maintain a support for the eye, which otherwise drops somewhat in the socket, producing a very objectionable form of diplopia. Upon incision of the growth the bleeding was very sharp. It came like pouring water from a glass. It was controlled by pressure of the finger until the actual cautery could be applied. I now show you the tumor preserved in Müller's fluid; and here, too, I hand you the bones removed in this case. These are the entire superior maxilla (save its orbital plate), the palate bone, the entire right turbinated bones, and about one-half of the malar bone. When the flap is stitched together over this great gap the deformity, as you see, is remarkably trivial. With a good dental prosthetical appliance, including a plumper for the cheek, I think no one at a distance of six feet would notice that anything had been done. Furthermore, if there should be a recurrence it can easily be reached through the free bony gap which is left. It will be a question for you gentlemen to decide for this patient, who to-morrow leaves the hospital, how best a half set of artificial teeth may be supported, whether part of the pressure could be brought to bear during mastication upon a broad leg of hard rubber carried up to rest against the orbital plate.

To replace the blood that was lost, while the subject was still on the operating-table, we injected into a vein at the bend of the elbow over two quarts of water as hot as the hand can bear, boiled and filtered, and containing about a heaping teaspoonful of common table-salt to the quart. Also a little strychnine was used hypodermically. The conjunction of these two forms the best means of preventing shock in grave operations in which the hemorrhage has been considerable. The result of this injection of warm salt-water was simply wonderful. Before we began to inject it the pulse was 140, and when we had finished it was down to about 80. Five years ago I spent my spare time during an entire winter experimenting, in the Columbia College Physiological Laboratory, on dogs in this matter, injecting after the withdrawal of blood hot salt-water in various degrees of strength and heat. In one instance, my assistant and myself both forgot to put any salt in the water, he supposing I had done it and I supposing he had; we injected plain warm water, and the result was that the dog died almost as quickly as if prussic acid had been given him. Professor Curtis, of the college, said death was due to the fact that plain water has the power to dissolve out the coloring matter from the red blood, thus destroying the red-blood cells. The addition of even six-tenths of one per cent. of common salt prevents this bad result.

I want to show you the latest improvement in the Nitze electric cystoscope. I got this while in Europe last summer. It is used for the illumination of the bladder in searching for possible stone or possible tumors, and to determine whether pus is entering from either ureter. We will use it here to illuminate the pharynx of this patient whose sarcoma operation I have just described, and you will be able to see with the clearness of day the walls of the cavity, and how much is left of the palate and interior of the nose. We will also, later, use this electric-light cystoscope to determine whether there is or is not turbid fluid, such as pus, in one antrum of Highmore. In illuminating the bladder that viscus is first washed out and filled with water.

This next patient from my clinic is a woman from Germany. She tells me that she has had trouble that involved the upper jaw for a year past. She says that she herself removed with her fingers a piece of flesh from the region of the jaw last summer. I find a condition which is in all probability a malignant epulis. I would like to have the opinion of you gentlemen who have had more experience in that line than I have. There is now some gingivitis, an accumulation of tartar, some inflammation, and some recession of the gums. Several of the teeth are loose. The epulis occupies the region of the alveolus corresponding with the right upper canine, and extending back to the first molar tooth, evidently springing from the sockets of the canine and the bicuspid teeth. We shall excise a small piece of the growth, and be guided in determining how much of the jaw to remove by the pathologist's report as to its nature.

[NOTE.—It proved to be a giant-cell sarcoma, and a large part of the jaw was accordingly excised at Professor Dawbarn's next clinic.]

Now I will give you some demonstrations on the cadaver. The first thing on your programme that would be of much interest is a demonstration of the section of the lingual nerve for cancer pain. When you have cancer of the tongue to deal with, an operation is always indicated right away. If the patient comes to you early, it will probably be sufficient to remove one-half of the tongue, as the growth commonly begins at the lateral edge; and if you advise the removal of only one-half of the tongue, the patient will perhaps submit; but if the patient comes to you at a later stage, and you have to suggest the removal of the whole of the tongue, he will be likely to object, and procrastinate until it is too late to hope for cure. In excision of the tongue most surgeons deem it wise to tie the lingual artery. There is practically no anastomosis between the two lingual arteries; as shown by the fact that if you inject one side of the tongue through one of the arteries, with some blue coloring matter, for example, one-half of the tongue will be absolutely blue and the other half will remain red. The same statement applies to the lymphatics. For that reason cancer does not readily spread from one side to the other, and the early removal of the affected side will often suffice. If one lateral half of the tongue is to be excised, the preliminary ligation of one lingual artery will permit an almost bloodless operation. Even in case the cancer has gone so far as to make a radical operation hopeless, the patient's life can be prolonged for months by tying the lingual of the affected side, or, better, both sides, and thereby depriving the growth of much of its nourishment. And suppose a case comes to you so late that the cancer has already involved the wall of the pharynx or the buccal surface, and a radical operation is hopeless. The patient is suffering agony; every time he moves the tongue it gives pain, and also every time he attempts to speak or swallow, and there is also excessive drooling of saliva. Here, by a simple operation that is absolutely safe and does not occupy more than ten seconds, this pain can be instantly relieved, without the taking of a particle of morphine, and that half of the tongue made so devoid of common sensation that a red-hot needle could be run through it without being felt. That operation is section of the lingual nerve. This we do by introducing within the mouth a curved, sharp-pointed bistoury, and making a cut against the ramus of the jaw running from a point three-quarters of an inch behind and below the grinding surface of the last molar tooth to that tooth. This cut divides the nerve with certainty.

Now, perhaps, you would like a demonstration of the operation for the removal of a considerable portion of the inferior dental nerve for the relief of tic douloureux, cases in which some doctors mistakenly advise the patient to have teeth out, and perhaps half the teeth in the lower jaw are removed without giving relief. I have seen several cases like that, as all surgeons have. When medicinal means have failed a section of the offending nerve should be removed at a reasonably proximal point. We make an incision downward along the rear border of the ramus, then along the lower border of the body of the lower jaw, the limits of the incision being the facial nerve above and the facial artery below. There is no artery of any importance in this region. This so places the scar that it will hardly be noticeable. Next the flap is reflected from this incision, including the periosteum with it, which leaves the outer surface of the ramus absolutely bare. Now, as you see, I chisel away a strip of bone an inch and a half long from the ramus, exposing the nerve, which is about the size of a knitting-needle, where it runs downward from the inferior dental foramen on its way to the teeth and the mental foramen. As long a piece of the nerve is excised as possible. Some surgeons trephine instead of chiselling, and then replace the trephine disk, which, of course, contains in its substance a corresponding piece of the nerve, and turn that disk a quarter of a circle, in order to prevent the nerve redeveloping by any possibility along its canal. The flap is now replaced, the divided periosteum being stitched at the point of incision in order not to lose the value of the masseter muscle, which was reflected with the periosteum from the ramus of the jaw in the operation, but is now restored to its former place.

We now know that bone deprived of its periosteum will still live; and in trephining from the skull it is customary, if an aseptic operation is being done, to save the trephine disk, wrapping it in a warm, moist, aseptic towel until the operation is finished, and then replacing the disk upon the *dura mater.* In about fifty per cent. of the cases this bone lives. If it happens to be bone having very little cancellous tissue, a still better chance for its life will be afforded by chiselling it into a number of fragments, as it is nourished by capillarity from the surrounding plasma, and the shorter the distance of the capillary movement the better the nourishment.

In surgery upon the skull-bones there are several ways in which to attack them. One is to cut our way through with a coarse bur in the dental engine. Another is the rongeur forceps. Our German friends almost always use chisels or gouges. That is all right in very cautious hands, but in brain-work it would be a trifle late to apologize after the gouge or chisel had slipped. And the constant jarring adds to the shock. If you use the chisel in the tic douloureux operation you press against the jaw so as to prevent as much as possible the jarring of the joint. Another way is to use a trephine, as I have described.

I will now show you the anatomy of the ligation of the lingual artery. I can demonstrate that ligation in two minutes, and—not to waste your time—will make a rapid sketch of its relationships in a minute and half more. [Dr. Dawbarn here illustrated his operation by a drawing by colored crayons on the black-board.]

This is one of the arteries that you can always depend upon to be where it ought to be. I have demonstrated it now eleven times on living subjects, and nearly three hundred times on the cadaver, in my operative surgery classes here; and I think those surgeons who have found anomalies in the course taken by this artery have simply not gone where they ought to have gone.

Now, I will ask you gentlemen, if you care to do so, to compare this dissection with the sketch that I have made on the black-board. Here is the posterior tendon of the digastric, here the anterior tendon of the digastric; here is the mylo-hyoid, which is drawn on the board in blue; here is a portion of the hyo-glossus, shown there in red. The twelfth nerve passes across and disappears under the mylo-hyoid, as in the sketch. Before I destroy this I will show you, by cutting through the hyo-glossus, the position of the artery running up towards the tongue. As you know, every artery in the dead subject looks flat. That is the only artery of any size in that region. It looks flat, even appears a little grooved; but during life, being full of blood, looks cylindrical. I will also show you with the needle the channel in the artery.

Nasal hemorrhage is something that we all ought to know how to treat, and it is inadvisable to wait more than a moderate time, trying the various remedies that may possibly succeed and are quite as likely not to succeed, before using means that are absolutely certain. Such a case (save the excessively rare contingency of hæmatophilia, which one may not see in a lifetime) can always be controlled in two minutes' time, to an absolute certainty. In mild cases we first gently plug the interior of the nose on the bleeding side with gauze, or a strip of handkerchief, soaked either in peroxide of hydrogen, a saturated solution of antipyrin, or dampened with a weak solution of cocaine up to five per cent. If these fail, then the scientific thing to do is to look for the cause of the bleeding, which is generally found in the anterior portion of the septum nasi, where, with a good light, blood will be seen trickling from the artery. By means of a metallic point, heated nearly red-hot and applied to the surface, it may be cauterized, and the bleeding checked almost at once. In the rare contingency where the bleeding-point is so far back as not to be seen with the aid of a forehead-mirror and reflected light we can always control the hemorrhage instantaneously by tamponning the nares. If the anterior naris is plugged, the posterior should be also, because otherwise the blood may continue to flow into the pharynx and be swallowed, the patient not realizing how much blood is being lost.

In all the works on surgery a Bellocq's canula is figured; I don't know why, because it is a needless instrument for this purpose. All that one needs for tamponning the anterior and the posterior naris is an ordinary soft-rubber catheter, such as is used for drawing urine. As you see, I now pass this through the nostril on the affected side, and presently its point appears in the pharynx. Seizing this with a pair of thumb-forceps, or a dressing-forceps, it is drawn out of the mouth; so that now one end appears through the nose and the other out of the mouth. Upon one of these ends we tie now a doubled piece of soft twine, say a yard long, and by completely withdrawing the catheter the twine is drawn into place; so that now we have the doubled twine entering the nose and escaping at the mouth. Upon the middle of this doubled twine we tie our posterior plug, folded from a piece of handkerchief, about the size of a large marble, and draw it into place. The anterior plug, much smaller, is now tied in place, aided by the doubled twine, and we are able to do this more tightly than we could have done with a single piece of twine. Now, the front and the back of the nose being absolutely closed, and connected by a tight string, all the blood that can flow will be the small amount which may fill one-half of the nose. The loose ends of string coming from the mouth and nose are tied together.

Such a tampon is left in place twenty-four hours, and the patient is warned not to try to blow out the blood-clot subsequently. This method of tamponning is much less annoying to the patient, uncomfortable though it is, than to insert a rubber or other sheath into the nose, and distend it with either water, air, or a gauze packing. The interior of the nose never was meant to be subjected to any other pressure than that of the atmospheric air. By the first method of tamponning the very sensitive turbinated bodies are not subjected to pressure; by the second method, which I have just said is extremely uncomfortable, they are subjected to a direct pressure, which is why I disapprove of it. The patient should be sitting upright. If you use a little cocaine spray, the operation is hardly annoying at all. As to how firmly we draw this rear tampon into place, a good rule is to draw it just for a moment with almost force enough to lift the patient's head by it. If peroxide of hydrogen is used, an objection to it is that it is irritating. In a certain case where I applied it for the purpose of dissolving the membrane in diphtheria, after I had used it two or three times my patient told me she preferred the diphtheria. Its irritating quality is due to a free acid, usually hydrochloric, which is put in by the makers to prevent the tendency of the excess of oxygen—which oxygen alone renders it of value as an antiseptic—from escaping. On account of this free acid it is very irritating to the membranes. If you will take a sample of any peroxide of hydrogen in the market, and touch a little of it to the conjunctiva of your eye, you will not wish to repeat the experiment. I make it a rule to neutralize all peroxide of hydrogen before use by putting a little ordinary soda bicarbonate, pinch by pinch, into the amount of peroxide which I intend to employ at that sitting until it no longer effervesces, when the free acid is neutralized. The peroxide itself will remain just as efficient as before for the purpose for which we employ it.

*Dr. McNaughton.*—Has not pyrozone the same objection?

*Dr. Dawbarn.*—I am not certain about free acid in pyrozone. It may be so. It could be readily ascertained by testing with litmus paper.

If you should meet with a severe case of nasal hemorrhage where even a soft-rubber catheter is not to be had, a twig from a willow-bush, or a stiff shoe-string, with twine and a bit of handkerchief, will answer the purpose for tamponning. The operation is even more easily done on the living subject than on the cadaver.

Œdema of the glottis is an accident that is so rapidly fatal if unrelieved, and yet so safely curable if treated properly, that every oral specialist should be familiar with the proper technique. It may be remembered that the minister to Russia, first appointed during President Harrison's administration, died at the Fifth Avenue Hotel, in this city, while *en route* to Russia, of a sudden development of this trouble, in the absence of a doctor. Had a doctor been on hand, his life could have been saved to a certainty. It is a condition in which two large dropsical bags, covered with mucous membrane, develop on either side at the top of the larynx, and nearly or quite meet in the middle, thereby obstructing the respiration. The pathological condition is an acute inflammatory distention, with serum, of the aryepiglottic folds. Now, if we take a curved, sharp-pointed bistoury, and wrap it with string or adhesive plaster down to within a quarter-inch of its point, we may safely guide it by the left forefinger, passed into the mouth and behind the tongue, which feels these bags of water, and cut them freely on either side, which brings immediate relief. There is no operation that is simpler than this one for the relief of œdema of the glottis.

The next operation will be one that is of interest to every one,

—namely, how to relieve that accident which is the source of occasional fatality, choking to death from "swallowing food the wrong way." It generally occurs while laughing or talking and at the same time endeavoring to swallow. A morsel of solid food enters the larynx, and is caught at the narrowest point,—the chink of the glottis (rima glottidis). If a medical man is present, it would be a lasting disgrace to him should the patient die, for relief may be obtained in a moment, with a single cut of a pen-knife in a perfectly safe region. This I will now demonstrate. You will observe that when the head is thrown back there are two prominences of the larynx, easily distinguishable by sight or touch. The uppermost is the so-called "Adam's apple," the lower is the inferior edge of the larynx, or cricoid cartilage. Between these is a small depression, into which always the tip of the finger may be made to sink. This depression, the crico-thyroid space, is where the cut should be made; guarding the pen-knife with the finger and the thumb so that only a quarter of an inch of the blade is permitted to cut, and with a single thrust, cutting horizontally, this crico-thyroid membrane is opened, and we enter the free air-space beneath. We cut horizontally because thereby, as I now show you upon this papier-maché model of the larynx, the space is wider transversely, and we thereby get more room, and also the crico-thyroid artery is less likely to be divided. At once the patient can inhale freely; and now through the cut we introduce some blunt instrument, like a lead-pencil, a hair-pin, or even a match, and dislodge upward the food which has caused the trouble; the patient can then cough it out. The operation is not tracheotomy, but laryngotomy. The only blood-vessel there is the little crico-thyroid artery, and that, if cut, can easily be controlled with the finger.

*Dr. Bogue.*—Can Dr. Dawbarn tell us something about that rather recent instance in which a cork was drawn into the trachea, and an operation performed for its removal?

*Dr. Dawbarn.*—That was a case in the practice of Dr. Fowler, of Brooklyn. A minister inhaled, while laughing and talking, a cork that he had just drawn from a medicine-bottle with his teeth; this cork passed beyond the larynx and into the windpipe, and he allowed some hours to elapse before calling in surgical assistance. At that time the cork was found lodged in one of his primary bronchi. Tracheotomy was done, and an attempt was made by Dr. Fowler, with a most ingenious instrument on the corkscrew principle, to extract the cork; but the mucous membrane had become so swollen from inflammation caused by this foreign body as to hold it firmly and render its extraction impossible. A portion of the cork did come away, but the remainder proved not removable, and the patient had to die with it in. Dr. Fowler attempted, after failing from the front, to attack the rear wall of the thorax, but he found it an extremely bloody and dangeous road, and had to give it up.

*Dr. Bogue.*—How far from the vocal cords do you cut in the laryngotomy which you have just demonstrated?

*Dr. Dawbarn.*—The vocal cords are almost mathematically in the middle of the larynx, measuring from top of thyroid cartilage to bottom of cricoid cartilage. The space we enter is well below the middle, and hence below the vocal cords, and below the food caught between them.

For the reduction of a dislocation of the lower jaw the usual plan is to depress the rear part of the lower jaw while lifting the anterior part, making the thumbs, wrapped in a handkerchief, a fulcrum. As soon as the articular condyle is forced below the eminentia articularis the temporal muscle will at once draw the lower jaw back to its normal place. Sometimes stout corks placed between the molar teeth are used instead of the wrapped thumbs. In one case in my experience, sent to me by a doctor in Bridgeport, the patient for some months had had an irreducible double forward dislocation of the lower jaw. Reduction had been attempted in the usual way by several excellent surgeons, among them Dr. Bacon, of New Haven, the patient being under general anæsthesia meanwhile; therefore I did not waste time in repeating these efforts. Instead, I made two incisions horizontally just below the zygoma, exposing the articular condyle in its false position; and then, by introducing a stiff instrument and using leverage against the tubercle of the zygoma, I succeeded in accomplishing the reduction. Of course, leverage had to be applied on both sides and through both cuts. The result was excellent, only two thread-like scars now showing that anything had been done. This operation is an exceedingly rare one. Hamilton "On Fractures and Dislocations," for instance, does not even allude to the possibility that a cutting operation may be needed in old cases of this difficulty.

*Dr. McNaughton.*—After three or four years had passed would it be possible to reduce such a dislocation?

*Dr. Dawbarn.*—It would be possible by means of a cutting operation, but not otherwise, because the temporal and other muscles of mastication would have become very much contracted in the new position of the jaw.

The scientific way to make a diagnosis of suppurative disease of the antrum is by the aid of a small electric light introduced within the mouth, the room being meanwhile dark. The tissues of the front part of the face are thereby made translucent and illuminated. On the affected side, however, the antrum being partially filled with a turbid fluid, pus, this somewhat obstructs the rays of light, and that side of the face will be comparatively dark. The difference is striking enough to be seen even from a considerable distance. The less scientific way of making the diagnosis is to have the patient first use the handkerchief thoroughly, and then lie in such a position that the antrum can drain by its natural opening into the nose. If, after a very few minutes with the head in this dependent position, a considerable amount of muco-pus may be expelled from the nostril on this side by blowing, the diagnosis is very probable; especially so if in conjunction with the presence of pain and tenderness and some swelling of the soft parts of the front of the face over the antrum. The treatment of this condition, which is often not recognized, and is attributed to neuralgia or to dental difficulties, is extremely simple,—one of the simplest in oral surgery.

There are several routes whereby the antrum may be entered and the pus drained. It will be evident that some artificial entrance must be made, because pus, like any other fluid, will not drain up hill, and the natural opening of the antrum is at the junction of its roof and inner wall, so it would be necessary for a man to stand on his head to drain it that way. We must therefore attack its floor for permanent drainage. One way in which this can be done is through the inferior meatus of the nose on the affected side. A second way is by extracting a tooth, and drilling up through its socket. This latter method has always seemed to me not only needless, but distinctly objectionable, inasmuch as a tooth that is even partially sound is too good a friend to be lightly sacrificed. The simplest way, and the best, in my judgment, is the one I now demonstrate for you,—namely, to make, under cocaine anæsthesia, an incision about an inch in length, extending from behind the canine tooth, backward along the junction of the gum with the cheek. The superior maxilla is then exposed just above the alveolar process. The bone covering the antrum is here so thin that the lightest tap of any steel instrument will readily penetrate it. It is almost as thin as a sheet of paper. And the cavity extends for such a distance above the bicuspid and molar teeth that no one could fail to find it. The ordinary dental engine would, with a coarse bur, give you entrance at once; or, if preferred, one may use, as I am now doing, a small gouge or chisel. An opening the size of the base of a lead-pencil is made, and through this the pus is washed out, some of the fluid escaping by the nasal passage into the nose. Preferably the peroxide of hydrogen is used once or twice daily for such irrigation. Following the washing, a short piece of rubber draining-tube is introduced, say half an inch in length, and is attached by a thread to the periosteum or the mucous membrane near the artificial opening. This tube is left in place until the discharge ceases. The advantage of this high incision, which nevertheless enters the floor of the antrum, is that the lip falls over this small tube in eating, and there will not be much likelihood of food making its way up into the antrum; whereas if a tooth is extracted, and the entrance forced through its socket, every movement of mastication tends to force food directly upward where we do not wish it to go. In certain very chronic cases we may have at a later stage to enlarge the bony opening, and with a sharp spoon scrape away the diseased mucous membrane, and sometimes the carious bone which in such cases may be found. The cavity is best when treated by packing daily, or on alternate days, with a long slender strip of iodoform gauze until all discharge has come to an end. I will now show you here what a large cavity the antrum is; with this bent probe you may examine its walls in all directions.

*Dr. Bogue.*—If the antrum is small, you simply make the opening farther back.

*Dr. Dawbarn.*—Yes. If not normally placed, the cavity is rather farther back than forward. I understood Dr. Bogue to say just before this lecture that he wished some questions answered in regard to cocaine. I will answer the questions now, if I can.

*Dr. Bogue.*—How do you use cocaine in this operation for antrum disease? After speaking of that please state a few general facts regarding the use of this drug in surgery.

*Dr. Dawbarn.*—I make a fresh solution every time, in the first place. You can get the tablets, half a grain or a grain each, and dissolve one in a little water. The trouble with a solution that you keep on hand is that it is not antiseptic at all. After it stands two or three weeks, if you examine it, you will find it reeking with microbes. Of course you can avoid that by adding carbolic acid in small amount (say one per cent.) to your solution, which will then keep well. Do not use bichloride of mercury, however, to prevent microbic development in your cocaine solution, for this is chemically incompatible with the cocaine, which will at once be precipitated as a white, milky deposit. I believe one grain of cocaine injected to be perfectly safe in a person in fair health, although there has been a case recorded where one grain was fatal; a case of a weak old lady, operated upon for ectropion. I have not heard of any fatal case from this dose excepting that. Occasionally you hear of some unfavorable symptoms from absorbing one grain, and it is well to use not more than two-thirds or three-quarters of a grain. When cocaine kills, it kills by contracting the blood-vessels of the brain. Respiration first fails from acute anæmia at the respiratory centre. The heart beats rapidly and weakly, but only stops after respiration fails. That being the case, if you will use something that will dilate the blood-vessels you will prevent and obviate the ill effects. For this purpose glonoin may be used, which is a one-per-cent. solution of nitro-glycerin and alcohol. You can get it in tablets. One to two drops of a one-per-cent. solution is a safe dose. By dissolving a little glonoin with the cocaine solution the ill-effect will be obviated. I should say that one or two minims would correspond to half a grain of cocaine. Perhaps the simplest way to prevent possible cocaine-poisoning is to give the patient a good drink of whiskey at the same time with the cocaine. Whiskey also tends to dilate the blood-vessels and to stimulate the heart. It is, moreover, somewhat anæsthetic also.

*Dr. Allan.*—In case you had this bad effect of cocaine, would it be proper to administer whiskey?

*Dr. Dawbarn.*—Yes. But after having the patient lie flat, the most rapid stimulant you can use is aromatic spirits of ammonia, one or two teaspoonfuls in a half glass of water. You need not be afraid of killing the person with that or with whiskey. Every one has noticed that in coryza you cannot breathe through the nose. In such a case if you snuff up a little cocaine solution it will immediately restore the breathing capacity, and in five minutes your nose will be as free as if there was never any such a thing as cold in the head. The nasal passages will be perfectly clear. Nevertheless, it is of no real benefit, because it only temporarily contracts the blood-vessels, and the swelling soon comes back worse than before, and lasts all the longer because you have done it. I only mention that as showing the marvellous power cocaine has of contracting the blood-vessels, and that is the way it kills. By the way, disease of the antrum almost always comes from coryza. A cold in the head continues for some time, the mucus gets thicker, the patient's cheek begins to ache, accompanied sometimes with fever and rigors.

*Dr. Allan.*—Does whiskey interfere with the action of cocaine?

*Dr. Dawbarn.*—No, not at all. It just occurs to me to say a word about pronunciation. The tendency is to pronounce this drug *cokane,*—a two-syllabled word, but it is properly pronounced co-ca-ine. A curious thing about this alkaloid is that its effects are only centrifugal; it travels centrifugally. That is why it is so ineffectual in dentistry, as you apply it in a cavity to the end of the nerve.

*Dr. Bogue.*—Is the effect on the lower portion of the nerve due simply to the drug being carried through the blood, or is there an absolute transmission in the nerve itself?

*Dr. Dawbarn.*—The latter is the case when the circulation is cut off temporarily. This is easily proved by injecting cocaine in contact with the ulnar nerve behind the elbow, the circulation being meanwhile shut off by a tourniquet. In a short while the little finger and half the adjacent one become completely numb. In dentistry you can benumb an upper central or lateral incisor by taking a little pledget of cotton and soaking it in a ten-per-cent. solution of cocaine and putting it in each naris close to the roots of the incisors, and leaving it ten minutes. You can then extract or fill the central or lateral incisor with almost no pain at all. I have tried it on myself, and know. It reaches the nerves of these teeth at a proximal point, so that the anæsthesia can travel centrifugally some distance. The nerve is benumbed, in other words, before it enters the root of the tooth.

*Dr. Bogue.*—Apropos of this use of the electric light for the purpose of making a diagnosis of disease of the antrum, it may be proper to say that light has also been used for determining the existence of death, a prize having been paid a doctor in Paris several years ago for a demonstration that whereas during life a bright light shining through the flesh between the closed fingers presents a pink hue from the circulating blood, after death the light shows no such pink hue, but an opaque appearance.

*Dr. Dawbarn.*—That is new to me. I had not heard of it. It is a very interesting fact, and I should like to try it.

On motion of Dr. Bogue, a vote of thanks was unanimously tendered to Dr. Dawbarn for his very interesting lecture and instructive demonstrations.

*Dr. Cook.*—Dr. Lord, our president, wished me to express his sincere thanks to Dr. Dawbarn for his kind attendance here this evening. Dr. Lord was compelled to leave early in the evening. Dr. Dawbarn has given us a most enjoyable lecture, and his demonstrations were the finest I have ever had the pleasure of seeing.

Adjourned.

S. E. Davenport, D.D.S., M.D.S.,
*Editor The New York Institute of Stomatology.*

